# A Clinical Study Comparing the Maxillary Anterior Tooth Shade and Fabricated Crown Shade of the Western Uttar Pradesh Population to Commercial Shade Guides

**DOI:** 10.7759/cureus.35719

**Published:** 2023-03-03

**Authors:** Tushar Sinha, Madhu Ranjan, Ujjal Chatterjee, Dharmendra K Sinha, Aditya Chaudhary, Anuraj Vijayan

**Affiliations:** 1 Department of Prosthodontics, Rajendra Institute of Medical Sciences, Ranchi, IND; 2 Department of Prosthodontics, Buddha Institute of Dental Sciences and Hospital, Patna, IND; 3 Department of Prosthodontics, Institute of Technology and Science (ITS) Dental College, Hospital and Research Centre, Greater Noida, IND

**Keywords:** shade tabs, lithium disilicate, light source, color, all ceramic

## Abstract

Background

It is essential for dentists and technicians to work together to fabricate and create restorations that are a "perfect" shade match for a particular person. Thus, the Vitapan 3D-Master tooth shade system (Vita Zahnfabrik, Germany) was created and put into use in order to improve the accuracy of shade-selection operations. The objective was to visually assess the color of the maxillary anterior teeth in male and female subjects from various age groups in Uttar Pradesh, India.

Materials and methods

There were 150 patients in total, and they were divided equally into three groups of 50: Group I, which included patients aged 18 to 30; Group II, which included patients aged 31 to 40; and Group III, which included patients aged 41 to 50. Ceiling-mounted fluorescent lighting fixtures with PHILIPS 65 D tubes (OSRAM GmbH, Germany) were installed. Three medical experts provided their opinions as part of this research. The maxillary central incisor was placed next to tabs of various shades, and the doctors' final assessment was based solely on the central one-third of the face. From each of the two sample sets, a total of 30 patients were selected. Once the crown had been made from the prepared tooth of the patient, it was colored according to two shade guidelines (Vita Classic and Vita 3D Master). The three clinicians matched the shade of the manufactured crown with visual shade guides. For shade matching, a modified United States Public Health Service (USPHS) standard was employed.

Results

The Chi-square test was used to compare categorical variables across groups. According to the Vitapan Classic shade guide, 26% of Group I participants matched the first Hue group (A1), 14% of Group II participants matched the first Hue group (A3), and 20% of Group III participants matched the second Hue group (B2). Master shade guide for Vita 3D 26% of Group I participants matched with the second value group (2M2), 18% of Group II participants matched with the third value group (3L 1.5), and 24.5% of Group III participants matched with the third value group (3M2). Eighty percent of people who were matched to Alpha scored for crowns made using the Vita 3D Master shade guide, while 94.1% of people who were matched to Charlie scored for crowns made using the Vitapan Classic shade guide in a comparison of the two shade guides.

Conclusion

The majority of the shades obtained from the Vita 3D master shade guide were found to be 1M1 and 2M1 in the younger patients, 2M1 and 2M2 in the second age group, and 3L 1.5, 3M2 in the older age group. In contrast, the Vitapan Classic shade guide revealed A1, A2, A3, B2, C1, D2, and D3 as the predominant shades.

## Introduction

Maintaining a patient's natural smile, which expresses their unique personality, is a difficult task for any prosthetic and restorative dentist. An aesthetic dentist's main duty is to determine and mimic the appearance of natural teeth. Due to their improved aesthetic, physical characteristics, and availability of new, cutting-edge materials, dental ceramics are now widely used in restorative dentistry. The fabrication and production of restorations by dental laboratories must therefore reflect a "perfect" shade match [1,2]. In dentistry, there are two methods for detecting color: visually and with instruments. By visual comparison of the patient's teeth to a reference shade guide, the shade is established. The main tools required to produce a color-matching result are computerized spectrophotometers and colorimeters, which measure color quantitatively. Due to their extreme accuracy, spectrophotometers cannot be used in clinical settings, which occasionally impairs the thinking of the technician and clinician [3].

It is highly improbable that the human eye could clinically distinguish between the minute differences in color between two similar objects. Because the determining factor for color is constant across a range of practitioners/clinics, visual color determination includes the judgment and assessment of the color of a patient's tooth with a common point of color reference. It is crucial to keep in mind that different people of different ages perceive colors differently, that some people have color vision problems (caused by diseases associated with a particular gender), that women tend to be more consistent than men, etc. This led to the development and introduction of the Vitapan 3D-Master tooth shade system (Vita Zahnfabrik, Germany) in an effort to improve and broaden the consistency and dependability of shade-selection methods [4,5,6].

The main objective of the current study was to visually identify the shade of the maxillary anterior teeth in various age groups of the male and female population of Uttar Pradesh, India. Then, using two different shade guides, an all-ceramic IPSE-Max lithium disilicate crown was created to restore a single maxillary anterior tooth.

## Materials and methods

Patients for the study were chosen from the Department of Prosthodontics & Crown & Bridge and Oral Implantology's outpatient clinic at the Institute of Technology and Science Dental College Hospital & Research Centre in Greater Noida, with approval from Institutional Review Board (IRB) (ITSDCGN/2018/001). A total of 150 patients participated in the trial, and all of them gave their written permission to participate. The following standards were applied: (a) there being at least one central incisor in the upper jaw; (b) the presence of one lateral incisor and one canine. The exclusion criteria were (a) individuals with significant attrition leading to incisal wear; (b) participants with any external/internal surface staining. The sample size was divided into the following groups, Group I consisted of 50 patients in the age group of 18-30 years, Group II consisted of 50 patients in the age group of 31-40 years, and Group III consisted of 50 patients in the age group of 41-50 years.

Luminous source

Three full-range, regular sunshine variety corrected Philips 65 D (OSRAM GmbH, Germany) fluorescent cylinders were installed in ceiling-mounted rich cylinder apparatuses to standardize the lighting. The fluorescent tube met the criteria for a standardized light source for choosing dental lighting shades.

Procedure for selecting a shade

The patients were seated up straight, with their mouths at the level of the clinician's eyes, and the doctor was at arm's length away. The clinicians' color vision was screened using the Ishihara test, but no cases of color blindness were found. Physicians only considered the center third of the face when making their final decision, and shade tabs were placed next to the maxillary central incisor.

Lithium disilicate all-ceramic crown shade matching

**Figure 1:**
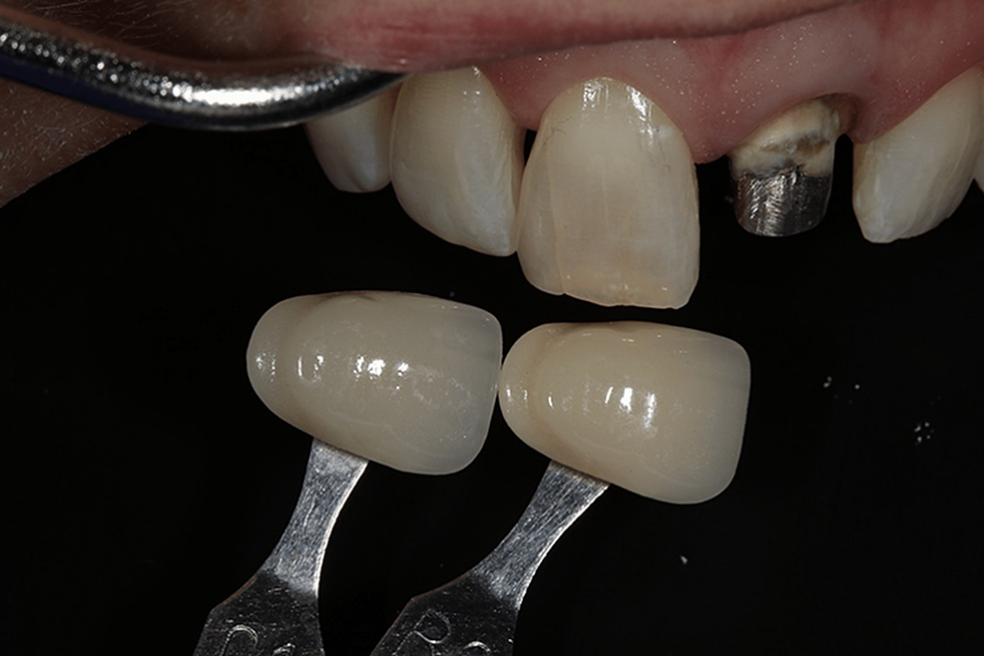
Vita shade guide with the tooth shade

From each sample group, 30 patients were chosen; 10 patients from each group needed cosmetic rehabilitation and an endodontically treated tooth for an all-ceramic crown on their maxillary anterior tooth. The same process was used to choose the shade before surgery. Temporary crowns were provided after tooth preparation was completed. Everybody received a pair of crowns. In order to accurately reproduce the color of the manufactured crown, both shade guidelines were used (Vita Classic and Vita 3D Master) (Figure 1).

The three clinicians matched the shade of the manufactured crown with visual shade guides. For shade matching, a modified United States Public Health Service (USPHS) standard was employed. Data were entered into an Excel spreadsheet and verified twice for accuracy. The analysis was conducted using IBM SPSS Statistics for Windows, Version 21 (Released 2012; IBM Corp., Armonk, New York, United States). After confirming the accuracy and reliability of the data, we used chi-square testing to check the statistical significance level for the inter-group comparison, and bivariate analysis was carried out for the paper. The means of two groups were compared using the Chi-square test for a set of categorical variables.

## Results

Twenty-six percent of Group I members matched to the first Hue group when using the Vitapan Classic shade guide (A1), 14% of individuals in Group II matched to the first Hue group (A3), and 20% of individuals from Group III matched to the second Hue group (B2). In Graph 2 under the Vita 3D master shade guide, 26% individuals in Group I matched to the second value group (2M2), 18% individuals in Group II matched to the third value group (3L 1.5), and 24.5% of individuals in Group III matched to the third value group (3M2). According to the USPHS score for Vitapan Classic, 40% of individuals in Group I were Charlie Score, 60% of individuals in Group II were Bravo Score, and 50% of individuals in Group III were Bravo Score (Table 1).

**Table 1:** Association and chi-square testing USPHS score for crowns fabricated by Vitapan Classic shade guide v/s age %: Percentage; n: number; USPHS: United States Public Health Service

UPSH Criteria for Vitapan Classic					
Age groups	Score A % (n)	Score B % (n)	Score C % (n)	Total	P-value
Group I	30 (3)	30 (3)	40 (4)	100 (10)	0.745
Group II	20 (2)	60(6)	20 (2)	100 (10)	
Group III	20 (2)	50(5)	30(3)	100(10)	
Total	23.3(7)	46.7(14)	30(9)	100(30)	

According to the USPHS score for the Vita 3D Master shade guide, 80% of individuals in Group I were Alpha Score, 80% of individuals in Group II were Alpha Score, and 60% of individuals in Group III were Alpha Score (Table 2).

**Table 2:** Association and chi-square testing USPHS score for crowns fabricated by Vita 3D Master shade guide v/s age %:  Percentage; n: number; USPHS: United States Public Health Service

USPHS criteria for Vita 3D					
Age groups	Score A % (n)	Score B % (n)	Score C % (n)	Total	P-value
18-30	80 (8)	10(1)	10(1)	100(10)	0.359
31-40	80(8)	20(2)	0 (0)	100 (10)	
41-50	60(6)	40(4)	0(0)	100(10)	
Total	73.3(22)	23.3(7)	3.3(1)	100(10)	

On comparison of the Vita 3D Master and Vitapan Classic shade guide, 80% of individuals matched to the Alpha score for crowns fabricated by the Vita 3D Master shade guide, and 94.1% of individuals matched to the Charlie score for crowns fabricated by the Vitapan Classic shade guide (Table 3).

**Table 3:** Association and chi-square testing intergroup comparison of crowns fabricated by Vitapan Classic and Vita 3D Master shade guide %: Percentage; n: number; USPHS: United States Public Health Service

USPHS criteria for VITA 3D					
USPHS classic	Score A % (n)	Score B % (n)	Score C % (n)	Total	P-value
Score A	50(3)	16.7(1)	33.3(2)	100(10)	0.025
Score B	71.4(5)	28.6(2)	0(0)	100(10)	
Score C	94.1(16)	5.9(1)	0(0)	100(17)	
Total	80(24)	13.3(4)	6.7(2)	100(30)	

## Discussion

Numerous studies that use both visual inspection and colorimetric analysis to pinpoint the most prevalent tones of the upper front teeth have been carried out, completed, and propagated [6,7]. Only a few studies have used this methodology on the Indian population to date, and of those that have, the majority of the research was carried out in South India, which has a different climate and geographic environment from the Northern parts, where the water bodies typically have a high fluoride content that affects the general population's dentition.

Khurana et al. in 2013 found a strong correlation between the two sets of data when we compared the shade of the maxillary anterior natural teeth of people of different ages in the Davangere region of India to those listed in commercially available shade guides [2]. A study was conducted in the Karnataka state of India by Rodrigues et al. in 2012 among various age and gender groups [7]. Therefore, it was intended for the current study to use two porcelain shade guides to perform a visual shade selection in order to identify the most common shade values in the Northern regions of India and to assess how accurate the shade guide was at predicting the aesthetic outcome of the restoration.

Although technology and digitalization have surpassed traditional and conventional methods in today's world, several studies have shown that visual shade selection is still preferable to digital methods for determining shade. Due to inherent calibration errors, it is claimed that the digital shade selection method using spectrophotometers is less reliable [8,9,10,11,12]. Vita 3D Master, according to Paravina et al. (2002), covers about 45% of the range of natural tooth colors [13]. The Vitapan Classic shade guide has sixteen tabs in total, with the letters A, B, C, and D standing for the four color categories (reddish brown, reddish yellow, yellowish, and reddish grey). Each category's color tabs are arranged in a chroma-based hierarchy. This shade chart was first published in 1960 and has since become the norm for the sector. Only about 11% of the visible surface area of real human teeth, according to the authors, is depicted by the colors in this shade chart. The hue-based, logically ordered arrangement of the shade tabs in the shade guide was lacking. It has been demonstrated that dental professionals all over the world are more dependable when using Vita 3D master shade guides for clinical shade matching.

According to Vafaee et al. (2012), when compared to Vitapan's traditional shade guide, Vita 3D Master significantly improved the shade matching exactness [14]. According to Ongul et al. (2012) [15], the Vita 3-D Master shade guide was found to have better color replication capabilities than the Vita Classic shade guide [16]. By a wide margin (56.67% to 0%), the Vita 3D Master shade guide system outperformed two other shade-matching systems and a colorimeter. It was discovered that the current study is consistent with this earlier research. For instance, Hammad et al. in 2003 demonstrated that inter-rater repeatability in shade choosing was significantly higher when using Vita's conventional shade guide [17]. The first and second value groups of the Vita 3-D master shade guide were matched by 70% of the participants in the first age group, according to the current study. Only 48% of samples in the second age group in the study were connected to the first and second value groups, while 52% were, respectively, to the third and fourth value groups. The major shades for the first age group were all found to have higher values in their respective hue groups, in accordance with Vitapan's classical shade guide, as opposed to the second and third age groups, which tended to have lower values for the major shades across the four hue groups. The most common shade values for this shade guide were discovered to be A1, A2, A3, B2, C1, D2, and D3 [13,18,19]. The current investigation also discovered secondary dentine deposition, which has been documented in the literature and results in teeth turning darker and more reddish with age. Younger people typically have lighter-colored teeth, and as people age, their teeth become darker [13]. Using shade recommendations for various age groups and orientations, Rodrigues et al. in 2012 conducted a study to find the best shade for maxillary and mandibular incisors [7]. This study was successful in halting the progressive aging-related discoloration of teeth. According to the Vita 3D expert who conducted this study, the most popular colors were 1M1 and 2M1 for the youngest patients, 2M2 and 2M1 for the middle-aged and elderly, and 3L1.5 and 3M2 for the oldest age group. These findings were consistent with those of other studies that were mentioned. The emotional nature of human variety perception due to visual impairment or variety discernment deserts, etc., are all factors that can negatively impact the nature of shade matching in the clinical setting. These differences in orientation, lighting conditions, metamerism, and eye fatigue that meaningfully affect the legitimate shade determination. The final shade coordination may be significantly impacted by a number of factors, such as tooth dehydration brought on by a prolonged clinical procedure and a change in the variety of the shade tabs following synthetic sanitation [7].

According to Fondriest (2003), shade selection should be done before tooth preparation and should be done in a specially designed black room equipped with color-corrected light [20]. He also recommended certain guidelines for the right lighting and environment for shade selection and the visual shade selection procedure. They recommended that the ideal light intensity should be between 2300 lumens and 95 or higher for CRI, so lights with a color temperature of 6500 K were taken into consideration for proper visualization. The current study had two observers stand five feet behind the primary investigator to make sure the values matched. The study group, including the primary scientists and observers, were all examined using Ishihara color charts to ensure that no one had any detectable color vision deficiencies. To avoid visual and image distortion brought on by the retina's cone receptors wearing out, choosing a new shade only took ten seconds for each shade. Even if the right hue is picked, it is still important to give the technician the exact information. Lithium disilicate (IPS e. max CAD by Ivoclar Vivadent), one of the most aesthetically pleasing ceramics, achieves this by having a glass matrix studded with needle-like lithium disilicate crystals, thereby minimizing the material's internal light scattering. The artificial teeth can blend in with the nearby natural teeth thanks to the chameleon effect and other visual characteristics. Due to this quality, we have chosen to use it to create the crown and verify the accuracy of the shade guide using the Modified Ryges or US Public Health Service standards [21,22].

Since this area is perpendicular to the light source and reflects the most light back to the viewer, the shade was chosen for the portion of the tooth that is in the center. Glare is more likely to occur on teeth with curved surfaces, such as canines and premolars. Flatter teeth, like central incisors, can provide more accurate shade selection because of their broad labial surface's uniform, diffuse lighting. With the desired ceramic layer thickness of 1.5 mm, lithium disilicate crowns were created from the chosen shades using both the Vitapan Classic shade guide and Vita 3D Master shade guide. Ghulman et al. in 2013 conducted a study to assess and compare the total color difference between natural teeth and manufactured crowns from three ceramic systems with different thicknesses of 0.8, 1.2, and 1.5 and discovered that E was insignificant when crowns were manufactured with 1.5 mm thickness, which was consistent with the present study, where it was observed that the shade of the manufactured crown matched with the chosen shade of all the samples [23].

According to Abou-Steit et al. (2019), lithium disilicate crowns have excellent patient satisfaction regarding color matching with the natural tooth when used in aesthetic regions [24]. According to Fasbinder et al. (2010), crowns made from computer-aided design (CAD)/computer-aided manufacturing (CAM) lithium disilicate showed no discernible color change after two years of use [25]. In their evaluation of posterior zone monolithic lithium disilicate crowns at 6 and 10 years, Rauch et al. (2018) reported USPHS alpha ratings [26]. Color differences in CAD/CAM lithium disilicate glass-ceramic restorations produced using were found by Chaiyabutr et al. (2011) [27]. Both options appear to be the same because of the material's optical properties, which allow the crown's final color to be influenced by the shade of the underlying tooth stump. Therefore, the current study did not include any teeth that were discolored.

It should be noted that in order for the ceramic prosthesis to have the same light reflectivity as the adjacent teeth, the surface texture, surface morphology, and anatomy of the natural tooth must be precisely replicated by the ceramic prosthesis. Therefore, even if the color of the restoration varies after the final selection of the shade accurately, differences in such important aspects can be easily appreciated and accepted [28,29]. As a result, choosing the right shade is an important but not the only factor in getting perfectly matched final prostheses. The dentist and lab must accurately communicate about shade, tooth architecture, and the subsequent precision of ceramic fabrication for the final cosmetic restoration to be well-received.

The limited selection of materials evaluated and the fact that each person will interpret color differently constitute the limitation.

## Conclusions

The conclusions that were drawn from the study were that 80% of people who were matched to Alpha scored for crowns made using the Vita 3D Master shade guide, while 94.1% of people who were matched to Charlie scored for crowns made using the Vitapan Classic shade guide in a comparison of the two shade guides. The majority of the shade obtained from the Vita 3D master shade guide was found to be 1M1 and 2M1 in the younger patients and 2M2 and 2M1 in the second age group followed by 3L 1.5 and 3M2 in the older age group, whereas the Vitapan Classic shade guide it was found to be A1, A2, A3, B2, C1, D2, and D3. The Vita 3D master shade guide produces a crown with the most acceptable shade match evaluated by the observer involved in the study using modified USPHS/ Ryges criteria compared to that of the Vitapan Classical shade guide. 
